# Advanced Platelet-Rich Fibrin (A-PRF) as Antibiotics Delivery System: In-Vitro Proof-of-Concept Study

**DOI:** 10.3390/ma18030570

**Published:** 2025-01-27

**Authors:** Giorgio Serafini, Alessia Mariano, Marco Lollobrigida, Luca Lamazza, Giulia Mazzucchi, Patrizia Spigaglia, Fabrizio Barbanti, Anna Scotto d’Abusco, Alberto De Biase

**Affiliations:** 1 Department of Oral and Maxillo Facial Sciences, “Sapienza” University of Rome, Via Caserta, 6, 00161 Rome, Italy; giorgio.serafini@uniroma1.it (G.S.); giulia.mazzucchi@uniroma1.it (G.M.); alberto.debiase@uniroma1.it (A.D.B.); 2 Department of Biochemical Sciences “Alessandro Rossi Fanelli”, “Sapienza” University of Rome, Piazzale Aldo Moro, 5, 00185 Rome, Italy; alessia.mariano@uniroma1.it (A.M.); anna.scottodabusco@uniroma1.it (A.S.d.); 3 Department of Infectious Diseases, Istituto Superiore di Sanità, Viale Regina Elena, 299, 00161 Rome, Italy; patrizia.spigaglia@iss.it (P.S.); fabrizio.barbanti@iss.it (F.B.)

**Keywords:** advanced platelet-rich fibrin (A-PRF), drug delivery systems, antibiotics, amoxicillin, metronidazole, antimicrobial resistance, oral surgery

## Abstract

Autologous blood centrifugation produces various forms of platelet concentrates widely used in tissue regenerative therapies due to their high concentrations of growth factors and abundance of autologous cells. Advanced Platelet-Rich Fibrin (A-PRF), introduced as a low-speed centrifugation product, contains an even higher concentration of growth factors, a greater number of cells, and a looser fibrin clot structure compared to previous Leukocyte and Platelet-Rich Fibrin (L-PRF). This study aims to assess the potential of A-PRF as a local delivery system for antibiotics. Different concentrations (0.5 mg/mL, 0.25 mg/mL, and 0.125 mg/mL) of injectable amoxicillin (AMX) and metronidazole (MTZ) were preliminarily tested for their impact on A-PRF clot formation, with 0.5 mg/mL selected for subsequent experiments. Blood samples from healthy volunteers were supplemented with antibiotics and centrifuged to form clots. Antibiotic-enriched A-PRF clots were immersed in phosphate-buffered saline (1x PBS) and analyzed at 24 h, 72 h, 7 days, and 14 days. AMX showed a consistent release (mean: 19.9 ± 4.8 ng/mL at 24 h) over 14 days, while MTZ demonstrated greater variability (mean: 12.8 ± 4.5 ng/mL at 24 h). AMX release remained constant over the 14-day period, with no significant variations among patients. In contrast, MTZ displayed a progressively lower release over time. Microbiological analysis revealed bacterial growth inhibition zones for *Fusobacterium nucleatum* (AMX: 23 mm, MTZ: 28 mm) and *Prevotella intermedia* (AMX: 34 mm, MTZ: 30 mm) at 24 h. These findings suggest that A-PRF can act as an effective local antibiotic delivery system, maintaining sustained antimicrobial activity and potentially reducing the need for systemic antibiotics.

## 1. Introduction

Antibiotics are the most common medications prescribed by general dentists and oral surgeons for both therapeutic and prophylactic purposes [1]. However, large-scale systemic administration of antibiotics can lead to several complications, including systemic toxicity, development of antibiotic resistance, and insufficient local drug concentration at the target tissue [2].

Administering drugs locally rather than systematically is a common way to decrease side effects and drug toxicity while maximizing a treatment’s efficacy [3]. Drug delivery systems (DDS) refer to engineered technologies designed and developed for the targeted delivery and/or controlled release of an active agent in the quantities and at the times necessary to optimize its therapeutic efficacy and safety, decreasing its systemic side effects and improving patient compliance [4]. Several carriers for topical antibiotic release have been proposed, including hydrogels, polymers, vesicular systems (liposomes), phospholipid-based microspheres, nanoparticles, and nano-hydroxyapatite/polyurethane composite scaffolds [5,6,7]. Most of them require complex and expensive biomolecular synthesis techniques, limiting their large-scale diffusion.

Autologous Platelet Concentrates (APCs) have been utilized over the years in various surgical fields, including orthopedics and oral and maxillofacial surgery [8]. The primary function of APCs is to support physiological tissue healing processes by locally releasing growth factors for up to 14 days [9]. APCs include two large families of biological products, Platelet-Rich Plasma (PRP) and Platelet-Rich Fibrin (PRF). PRP involves several centrifugation phases and requires anticoagulants and activators [10], while PRF is obtained with a single centrifugation and does not require specific blood manipulation [11]. Advanced Platelet-Rich Fibrin (A-PRF), introduced in 2014 by Ghanaati et al. [12], is obtained by decreasing the revolutions per minute (rpm) and increasing centrifugation time. This slower centrifugation increases neutrophil granulocytes in the distal part of the A-PRF clot, contributing to the differentiation of monocytes into macrophages. Monocytes, macrophages, and growth factors within A-PRF can influence bone and soft tissue regeneration. An alternative clinical application of APCs has been suggested, including the liquid form of PRF, as an advanced local delivery system [13,14] for small and large biomolecules, such as antibiotics, corticosteroids, and other therapeutics. This approach could modulate immune response and reduce the likelihood of foreign body reactions within host tissues post-injection observed with other delivery systems [15]. This study expands on the current literature by demonstrating the potential of A-PRF as an innovative drug delivery system. Unlike synthetic carriers, A-PRF is biocompatible, cost-effective, and derived from autologous sources, reducing the risk of immunogenic reactions. These features make it a promising alternative for localized drug delivery, particularly in resource-constrained settings or for patients with contraindications to systemic antibiotics. To date, limited data are available on the release profile of commonly used drugs from PRF clots. Amoxicillin (AMX) and metronidazole (MTZ) are among the most commonly used antibiotics for the treatment and prevention of odontogenic infections [16,17,18]; therefore, their potential application with delivery systems is of interest.

The aim of this in vitro study was to evaluate, through biochemical and microbiological analysis, the effect of adding AMX and MTZ to A-PRF, analyzing their release profile over a 14-day period and their growth inhibitory capability against two periodontal bacterial pathogens. Furthermore, the bacterial growth inhibitory capability of the antibiotic-containing PBS samples against strains of *Fusobacterium nucleatum* and of *Prevotella intermedia* was also evaluated.

## 2. Materials and Methods

### 2.1. Patient Selection

According to the Declaration of Helsinki of 1975, revised in 2013, on medical protocol and ethics, the Ethics Committee for Human Research of Sapienza University of Rome approved blood collection for experiments related to PRF (approval number: 906/19). A written informed consent was obtained from all study participants before the treatment. Five healthy volunteers were included in the study.

The exclusion criteria were as follows:

- Young people (under the age of 18).
- Volunteers with the willingness and ability to give their writing informed consent.
- Absence of contraindications to blood sampling.
- Use of systemic antibiotics in the past six months.
- Drug therapy with non-steroidal anti-inflammatory drugs (NSAIDs) for a long time.
- Antiplatelet and/or anticoagulant medication therapy.
- Blood disorders (i.e., coagulopathies, thrombocytopenia).
- Pregnancy or lactation.

### 2.2. Biochemical Tests

Preliminary tests were performed to investigate the effects of three different concentrations (0.5 mg/mL, 0.25 mg/mL, and 0.125 mg/mL) of injectable AMX and MTZ on the formation of A-PRF clots. A final 0.5 mg/mL concentration of the antibiotics was used for the subsequent tests. Ten milliliters of peripheral venous blood were twice sampled from 5 healthy volunteers. One milliliter of AMX and one of MTZ stock solutions, containing 5 mg/mL antibiotics, were added to the first and second tubes, respectively, obtaining a final concentration of 0.5 mg/mL of each one.

The antibiotic-enriched samples were then centrifuged for 14 min at 1500 rpm using an Intraspin™ centrifugation device (33° rotor angulation, 50 mm radius at the middle of the tube, 80 mm at the maximum, and 40 mm at the minimum) [19] (Intra-Lock, Boca-Raton, FL, USA) to obtain A-PRF clots. The glass tubes did not contain anticoagulants or additives. The obtained clots were then separated from the erythrocyte phase and immersed in a small volume (300 µL) of phosphate-buffered saline (1x PBS) to evaluate the antibiotic release after 24 h, 72 h, 7 days, and 14 days. At each collection time, 300 µL of antibiotic-containing PBS was retrieved and stored at −20 °C, while the volume of PBS withdrawn was replaced with 300 µL of fresh PBS. Enzyme-linked immunosorbent assay (ELISA) tests (Elabscience Bioinnovation Inc., Wuhan, China) were then performed on the antibiotic-containing PBS in 96-well plates, where all reagents and sample aliquots were added.

### 2.3. In Vitro Pharmacokinetic Analysis

In order to describe the pharmacokinetics of AMX and MTZ release from L-PRF over time, in vitro release profiles were correlated with four kinetic models: zero order (cumulative amount of drug released vs. time), first order (log cumulative % of drug remaining vs. time), Higuchi (cumulative % of drug released vs. square root of time), and Hixon–Crowell (cube root % of drug remaining vs. time) release equations (Table 1). For each model, the r^2^ and the kinetic constant have been calculated [20,21].

### 2.4. Microbiological Analysis Method

Microbiological analysis was carried out using antibiotic-enriched PBS samples, obtained through the immersion of PRF for 24 h, and *F. nucleatum* clinical isolate AP05/97 and *P. intermedia* ATCC 25611 (Leibniz-Institut, DSMZ-Deutsche Sammlung von Mikroorganismen und Zellkulturen GmbH, Braunschweig, Germany), as representative of two bacterial pathogens cause of periodontal diseases. *F. nucleatum* and *P. intermedia* were selected as representative pathogens frequently implicated in periodontal and oral infections. These bacteria are established models in studies evaluating antimicrobial efficacy, providing a focused and clinically relevant framework for this proof-of-concept investigation.

Bacterial strains were grown on blood agar (BA) plates supplemented with 5% sheep blood cells, 5 mg/L hemin, and 0.5 mg/L vitamin K, and, after 24 h of incubation in an anaerobic atmosphere, the cultures were stored in cryotubes at −80 °C for subsequent analysis [22,23].

The minimum inhibitory concentration (MIC) values for AMX and MTZ were determined using the agar dilution method on pre-reduced Brucella blood agar (BBA) plates supplemented with 5 mg/L hemin, 1 mg/L vitamin K1, and 5% defibrinated sheep red blood cells, as recommended by the Clinical and Laboratory Standards Institute (CLSI) [24,25]. The breakpoints used for the interpretation of the MIC values were those recommended by the CLSI guideline and the European Committee on Antimicrobial Susceptibility Testing (EUCAST) [26,27]. The *B. fragilis* ATCC 25285 was used as a control strain.

The bacterial growth inhibitory capability of antibiotic-containing PBS samples was tested by disk diffusion using BBA plates. Briefly, after 48 h of incubation at 35 °C in an anaerobic chamber (90% N_2_, 5% CO_2_, 5% H_2_), a suspension at 1 McFarland of each bacterial strain was prepared in Brucella broth added with 5 mg/L hemin. Each bacterial suspension was uniformly inoculated onto a plate by streaking with a swab containing the inoculum. The paper disk (Oxoid™ Blank Antimicrobial Susceptibility disks), soaked with 10 µL of each antibiotic-containing PBS sample, was centrally placed on the surface of an inoculated and dried plate. After 24 h of anaerobic incubation, the diameter of the inhibition zones around the disks was measured, as recommended by international guidelines [28,29]. Bacterial strains were also tested using paper disks containing 10 µL of AMX and MTX, respectively (Oxoid™ Amoxycillin Antimicrobial Susceptibility disks; Oxoid™ Metronidazole disk). The quality control *B. fragilis* ATCC 25285 was included in each experiment to assess reproducibility. A disk soaked with 10 µL of PBS was used as a negative control. Each experiment was carried out in triplicate.

### 2.5. Statistical Analysis

The statistical analysis was performed using a nonparametric one-way ANOVA (Kruskal–Wallis test) and post hoc Mann–Whitney *U* (MWU) test (adjusted by the Bonferroni correction for multiple tests), applied on the original data to obtain a comparison between groups at each time point. The data were plotted in histograms using Prism 5.0 software (GraphPad Software, San Diego, CA, USA).

## 3. Results

Five systemically healthy volunteers were included in the study. The gender distribution of patients was 4 males (80%) and 1 female (20%), with a mean age of 28.8 years (ranging between 24 and 35 years). Two samples of 10 mL of peripheral venous blood were collected from each volunteer. The A-PRF clots obtained after centrifugation were semi-solid and yellowish in appearance, with an average diameter of approximately 10 mm and a length of 25 mm, as assessed on sterile graph paper. Their consistency was gel-like, with a homogenous and loose fibrin network structure. The clots were carefully separated from the erythrocyte layer using sterile instruments to preserve their integrity. No significant variations in size or appearance were observed between samples.

### 3.1. Biochemical Analysis

**Figure 1:**
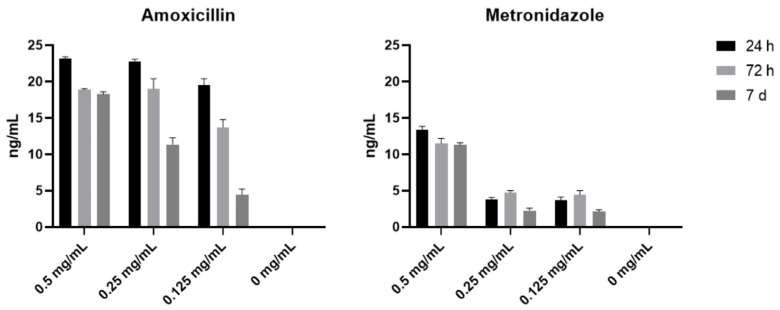
Antibiotics’ release by A-PRF. The release of AMX and MTZ in PBS was measured using ELISA at 24 h, 72 h, and 7 days after collection. Three concentrations were analyzed: 0.5 mg/mL, 0.25 mg/mL, and 0.125 mg/mL, and 0 mg/mL represented the negative control, A-PRF without antibiotics. Results are expressed as the means ± SD of data obtained by three independent experiments.

Preliminary tests were carried out to find an optimal antibiotic concentration not interfering with the physical properties of the PRF clot. Three different concentrations of injectable AMX and MTZ were assessed (0.5 mg/mL, 0.25 mg/mL, and 0.125 mg/mL). As far as the release of antibiotics is concerned, the amount of AMX released in PBS was always higher than that of MTZ and did not show significant differences among the three concentrations analyzed in the first 24 h after A-PRF clot collection. Greater differences in the amount of AMX released were observed after 72 h and after 7 days from sampling, showing a higher release in the sample containing 0.5 mg/mL AMX concentration. Unlike AMX, the release of MTZ from A-PRF clots was more influenced by the initial concentration, showing a significantly higher release at 0.5 mg/mL MTZ concentration compared to the 0.25 mg/mL and 0.125 mg/mL concentrations (Figure 1). The negative control, A-PRF without antibiotics, was represented in the histograms (0 mg/mL).

The concentration of 0.5 mg/mL resulted in a higher release of both antibiotics from A-PRF clots and, for this reason, it was used for subsequent tests. Based on the results obtained at 7 days, it was decided to extend the subsequent analysis to up to 14 days.

**Figure 2:**
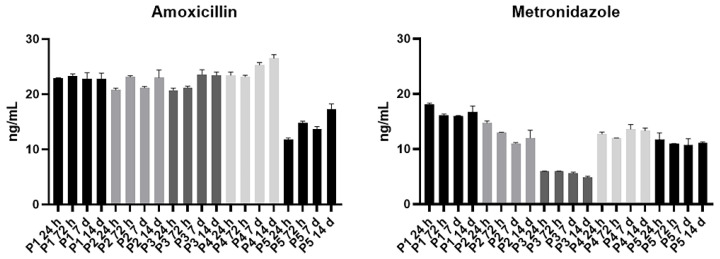
Antibiotics’ release by A-PRF. The release of the antibiotics AMX and MTZ in PBS was measured using ELISA at 24 h, 72 h, 7 days, and 14 days after sample collection from five volunteers. A total of 0.5 mg/mL of each antibody was added in A-PRF clots.

**Figure 3:**
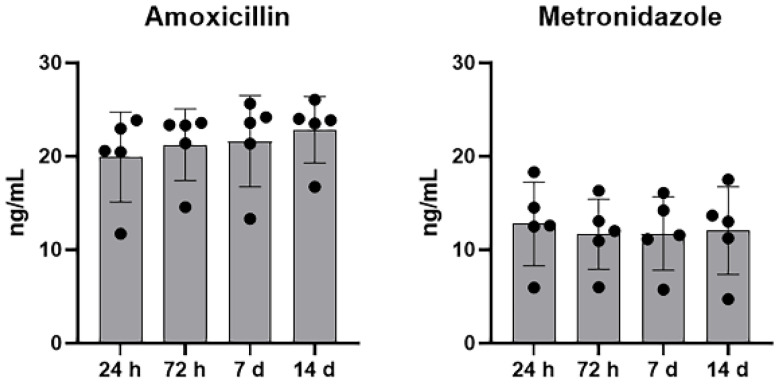
Antibiotics’ release by A-PRF. The cumulative concentration of each antibiotic, considering the five samples, was calculated at each analyzed time point. Black circles represent individual values of participants. Results are expressed as the means ± SD of data obtained by three independent experiments.

Similarly to the preliminary analysis, a greater amount of AMX was released from A-PRF clots compared to MTZ. This release was consistent over time and did not show significant variations among healthy volunteers. Differently, MTZ exhibited greater inter-individual variability but still showed a consistent release observable up to the 14th day (Figure 2 and Figure 3).

### 3.2. Pharmacokinetic Analysis

The pharmacokinetics of AMX and MTZ in vitro release from A-PRF are presented in Table 2. Analyses were performed up to 14 days, and cumulative antibiotic release and release measured in percentage (%) have been calculated. The cumulative AMX release after 14 days was 102.73 ± 20.17 ng, whereas the MTZ release was 57.84 ± 19.89 ng. The release in % of both antibiotics was less than 1% of the antibiotic concentration added into the whole blood sample.

The antibiotic release kinetics were described by fitting the biochemical analysis data into various kinetic models, and the kinetic constant (K) and regression coefficient (R^2^) were determined, as indicated in Table 2. Zero order, first order, Higuchi, and Hixson–Crowell were used as models to describe the drug release from A-PRF and to compare the differences in the release of the two antibiotics over time. Considering that the zero-order kinetic is only a function of time and is independent of antibiotic initial concentration, the kinetic constant, K_0_, indicates the differences in release between AMX and MTZ. These data confirm that the AMX release was higher than the MTZ release, as stated above.

Regarding the first order release analysis, the K value for the two antibiotics was almost the same, K_AMX_ = 0.030 and K_MTZ_ = 0.031. Considering that K is measured on the percentage of cumulative remaining antibiotics, the data showed that both antibiotics had quite the same remaining amount. The Higuchi model depends on the square root of time and is based on Fickian diffusion, independently of initial antibiotic concentration, as well as the zero-order model. In our analysis, once again, the AMX constant rate was higher than that observed for MTZ. The Hixson–Crowell model is based on the cubic root of the remaining antibiotic amount, and a very low difference can be observed in K_HC_, suggesting a low molecule release over time. Finally, in the zero order, first order, and Hixson–Crowell model, a low value of the coefficient of correlation R^2^ was found; thus, the release does not follow the principle of these mathematical models. The graphical representation of cumulative % of drug release against the square root of time represented that AMX and MTZ release from A-PRF was perfectly following the Higuchi drug release model, as the antibiotics release profile was very close to the regression line, and there was the highest value of the coefficient of correlation (R^2^ = 0.9939 for AMX and R^2^ = 0.9952 for MTZ) (Table 2).

### 3.3. Microbiological Analysis

**Figure 4:**
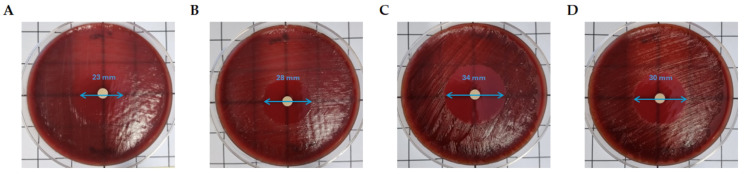
Results of disk diffusion test on *F. nucleatum* AP05/97 203 and *P. intermedia* ATCC 25611 using disks soaked with PBS samples containing antibiotics released from A-PRF clots. *F. nucleatum* growth inhibition with a disk soaked with 10 µL of PBS samples containing AMX (**A**) and MTZ (**B**). *P. intermedia* growth inhibition with disks soaked with 10 µL of PBS samples containing AMX (**C**) and MTZ (**D**). The inhibition zone diameter is indicated with blue arrows.

The MIC values obtained using the agar dilution method for *F. nucleatum* AP05/97 and *P. intermedia* ATCC 25611 are shown in Table 3 and Figure 4 and indicate that the two strains were susceptible to both the antibiotics tested. The *B. fragilis* 25,285 control strain shows MIC values of 0.5 ng/μL when MTZ was tested and 0.25 ng/μL when AMX was tested.

The results on the bacterial growth inhibitory capability of the antibiotic-containing PBS samples, collected at 24 h, indicated that the samples tested were able to inhibit the growth of both *F. nucleatum* AP05/97 and *P. intermedia* ATCC 25611.

## 4. Discussion

A drug delivery system (DDS) is defined as a technological formulation or a tool for the introduction of a drug molecule within the body by controlling the rate, extent, and location of drug release [30]. Drug delivery systems offer several key advantages, including increased local drug concentration, prolonged retention time, enhanced bioavailability, and reduced drug distribution in non-target organ/tissue [5,31]. These positive findings are especially significant in tissues with limited blood supply, such as bone tissue. In contrast, systemic administration distributes antibiotics not only to the intended site of action but also to various organs (e.g., lungs, tonsils, prostate, middle ear mucosa) [32]. As a result, prolonged antibiotic therapies are often required to maintain local concentrations above the minimum inhibitory concentration (MIC) required to effectively inhibit pathogens [33]. While localized delivery strategies have shown considerable advancements, their clinical translation faces several hurdles, including safety, reproducibility, and patient compliance. Most nanostructured and scaffold-based delivery systems rely on synthetic materials, with safety and reproducibility in large-scale production being primary concerns [34]. To investigate the in vivo efficacy of local drug delivery systems, numerous studies employ animal models, although the use of humanized models is increasingly advocated in research [35]. In this context, the design of multifunctional wound dressings is essential to prevent infection and promote healing. Compared to synthetic delivery systems, A-PRF offers significant technological advancements by integrating the regenerative properties of PRF with the controlled release of antibiotics. PRF represents a unique system characterized by biocompatibility and biodegradability, along with growth factors and peptides that support tissue regeneration [36]. The high concentration of growth factors and cell availability make PRF an ideal system for tissue engineering, as well as for hard and soft tissue regeneration. PRF is commonly used in dentistry for improving wound healing, in periodontology, for orthodontic tooth movement, and in endodontics, for bone regeneration [37]. The rationale for using PRF in oral surgery lies in its ability to facilitate the regeneration of both soft and hard tissues. Moreover, the three-dimensional fibrin matrix could act as a carrier for adjunctive substances and drugs, functioning effectively as a drug delivery system. Considering that infection is the most common postoperative complication in oral surgery, combining PRF with antibiotics in a single drug delivery system represents a promising strategy for enhancing treatment outcomes in this field.

To the best of the authors’ knowledge, this study is the first to investigate the potential of A-PRF as a drug delivery system. While several previous studies have examined the combination of conventional PRF with antibiotics, such as metronidazole, clindamycin, penicillin, vancomycin, teicoplanin, gentamicin, and amikacin, to target oral pathogens and enhance the healing process, our work uniquely explores the advanced formulation of PRF using a distinct centrifugation protocol.

Polak et al. [38] investigated the potential of PRF as a sustained local delivery system for various antibiotics, including metronidazole (MTZ) at 5 mg/mL, clindamycin at 150 mg/mL, and penicillin at 1 mU/mL. The antibacterial efficacy of antibiotic-enriched PRF was assessed against *S. aureus* and *F. nucleatum*. PRF with antibiotics showed significant antibacterial activity, with MTZ and clindamycin exhibiting greater efficacy against *F. nucleatum* compared to penicillin. Against *S. aureus*, MTZ was less effective than clindamycin and penicillin. PRF clots were tested over 96 h, consistently demonstrating antimicrobial activity without antibiotics compromising the physical properties of PRF. However, unlike our study, Polak et al. investigated PRF clots obtained using conventional centrifugation speeds (2700 rpm). Moreover, both clots and membranes were tested directly on agar plates, leading to a potential confounding factor, as the intrinsic antibacterial properties of the membranes (due to the presence of leukocytes) could contribute to the observed inhibition zones. In our study, we employed a medium to ensure that the inhibition zones observed were exclusively due to the antibiotic released by the clots.

In the study of Bennardo et al. [39], PRF was combined with gentamicin, linezolid, and vancomycin and analyzed at multiple time points (1, 2, 3, and 4 days). PRF was prepared using the L-PRF (Leukocyte- and Platelet-Rich Fibrin) protocol, and its antimicrobial effect was tested against *E. coli*, *P. aeruginosa*, *S. mitis*, *H. influenzae*, *S. pneumoniae*, and *S. aureus*. In that study, the addition of vancomycin led to significant changes in the physical properties of PRF or inhibited PRF formation altogether. This was attributed to the spontaneous aggregation of red blood cells induced by vancomycin at concentrations exceeding 3.0 mg/mL. Antimicrobial release from platelet concentrates was observed throughout the entire experimental period.

In accordance with previous studies, our results clearly demonstrate that PRF can be used as DDS combined with different antibiotics. In particular, the antimicrobial effect of both AMX and MTZ, two of the most commonly used antibiotics in the treatment of periodontitis, was demonstrated for overall time on two predominant bacterial species implicated in periodontal diseases: *F. nucleatum*, a well-known pathogen that is capable of invading human gingival epithelial cells, remaining viable inside host cells [40], and *P. intermedia*, which is frequently found in dysbiotic biofilms of periodontal diseases and other conditions, contributing to oral inflammatory processes [41].

Knafl et al. [42] reported that teicoplanin and amikacin released from a PRP-antibiotic co-delivery system showed antimicrobial in vitro effects for almost seven days. In our study, antibiotic release was steady up to 14 days in accordance with data reported by Rafiee et al. [43], although a progressively lower release of MTZ was observed over time compared to AMX. In the study by Rafiee et al. a mix of three antibiotics (TAP)—metronidazole, ciprofloxacin, and minocycline—was combined with liquid PRF (I-PRF). I-PRF worked as a scaffold for evaluating drug release profiles. Interestingly, MTZ was the only antibiotic detectable on day 28. Authors have hypothesized that MTZ forms a tighter bond to the I-PRF surface, which results in a more sustained release. In our study, MTZ profile release showed inter-individual variability greater than AMX. MTZ release was influenced by the initial concentration. At a concentration of 0.5 mg/mL, the higher release has been observed. These findings are consistent with a previous study by Siawasch et al. [44], where the authors reported that MTZ release was highest at the initial time point (4 h), with statistically significant dose-dependent differences. In that study, the antimicrobial activity was evaluated against *P. gingivalis*, *P. intermedia*, and *F. nucleatum* using the agar diffusion test. The incorporation of MTZ into PRF resulted in a significantly greater inhibition zone compared to control membranes. The inhibition observed with L-PRF membranes modified with MTZ was also dose-dependent. Interestingly, the volume of MTZ added did not exceed 0.5 mL to prevent physical alterations in L-PRF membranes, with a concentration of 2.5 mg/mL. Finally, the authors administered both MTZ and amoxicillin (AMX) orally 2 h prior to blood collection, yielding results comparable to those achieved with the addition of drugs to the tubes. Considering the disadvantages of systemic drug administration, the authors state that the addition of local antimicrobials to L-PRF before centrifugation could represent the most suitable drug delivery option. Our data on AMX release and antimicrobial activity cannot be compared because of the different addition protocol of AMX and, as already observed for the study of Polak [38], the membranes were directly tested on blood agar plates.

Finally, the observed higher release concentration of AMX compared to MTZ may be attributed to several factors related to the physicochemical properties of the antibiotics and their interactions with the PRF matrix. AMX is more soluble in aqueous environments than MTZ [45], which likely facilitates its diffusion through the PRF matrix, resulting in a higher release rate. Additionally, the binding affinity of AMX to the components of PRF, such as fibrin and platelets, may differ from that of MTZ. If AMX binds less tightly to the PRF matrix, it would be released more readily, contributing to the observed difference in concentration. Furthermore, AMX’s greater hydrophilicity compared to MTZ may enhance its release from the predominantly aqueous PRF matrix. We hypothesize that these factors collectively may explain the disparity in the release profiles of the two antibiotics, though future studies should investigate these mechanisms further to optimize antibiotic delivery systems using PRF matrices.

Moreover, through in vitro pharmacokinetic studies, it has been shown that drug release from the A-PRF follows the mathematical model of Higuchi. This result means that the release follows Fick’s law based on diffusion principles.

Overall, our findings demonstrated that PRF can act as a drug delivery system for local release of AMX and MTZ and that the amount of released antibiotics is effective against two of the most common pathogens associated with odontogenic infections at all tested time points. The potential applications of A-PRF as a drug delivery system extend to various surgical and regenerative fields, including oral surgery, maxillofacial reconstruction, and wound management. Future in vivo studies are critical to validate its safety, efficacy, and potential to reduce systemic antibiotic use. These studies should assess not only the pharmacokinetics and pharmacodynamics of antibiotic release in living systems but also the effects on tissue regeneration, immune response, and long-term biocompatibility. Randomized clinical trials will be essential to confirm its utility in managing postoperative infections and improving patient outcomes. Furthermore, future studies should explore the incorporation of other therapeutic agents, such as anti-inflammatory drugs or growth factors, to broaden clinical applications, as well as additional antibiotic classes, such as macrolides and lincosamides, particularly for patients with penicillin allergies.

This study has some limitations: it has been conducted on a few healthy volunteers that may not be representative of the whole population with respect to PRF characteristics; the use of only two commercial bacterial strains may limit the generalizability of the study results in relation to the polymicrobial nature of most infections; finally, the potential effects of adding antibiotics on the physical characteristics and therapeutic properties of PRF warrant further investigations. Additional research, randomized controlled clinical trials, and in vivo experiments are necessary to validate the promising results obtained in this study.

## 5. Conclusions

The results obtained from this study suggest that A-PRF could serve as a local antibiotic delivery system for AMX and MTZ at effective concentrations. The application of A-PRF supplemented with antibiotics following oral surgery procedures may reduce the risk of postoperative infection and diminish the need for systemic antibiotic therapies, preserving the inherent therapeutic properties of PRF itself. These findings could potentially expand the application of PRF combined with antibiotics for the treatment of various oral pathologies and for preventing infectious complications in regenerative bone surgery.

## Figures and Tables

**Table 1:** Drug-release kinetic models and equations.

Model	Equation	Terms Used in Equation
Zero order	Q_0_ − Q_t_ = K_0_t	Q_t_ = quantity of drug released at time t; Q_0_ = initial amount of drug in solution; K = kinetic constants; t = time.
First order	Log (Q_0_) − Log (Q_t_) = Kt/2.303	
Higuchi	Q_t_ = K_H_T^1/2^	
Hixon-Crowell	Q_0_^1/3^ − Q_t_^1/3^ = K_HC_t	

**Table 2:** Comparative kinetic parameters of antibiotic release from A-PRF.

Antibiotic	Model							
	Zero Order		First Order		Higuchi		Hixson–Crowell	
	R^2^	K_0_	R^2^	K	R^2^	K_H_	R^2^	K_HC_
Amoxicillin	0.9415	0.3035	0.8965	0.0300	0.9939	5.6043	0.9416	0.0390
Metronidazole	0.9459	0.1696	0.8942	0.0318	0.9952	3.1554	0.9460	0.0392

**Table 3:** Antibiotic susceptibility tests. ^a^ Medium values obtained from three independent experiments.

	Agar Dilution MICs (ng/µL)		Disk Diffusion Zone of Inhibition (mm) ^a^ Using			
	AMX ^b,c^	MTZ ^b,d^	AMX Disk (10 µg) ^e^	MTZ Disk (5 µg) ^f^	10 µL AMX-Containing PBS Samples ^e^	10 µL MTZ-Containing PBS Samples ^f^
*F. nucleatum* AP05/97	0.064	0.125	44	35	23	28
*P. intermedia* ATCC 25611	0.064	1.0	60	40	34	30

^a^ Medium values obtained from three independent experiments. ^b^ AMX: amoxicillin; MTZ: metronidazole. ^c^ The AMX susceptibility breakpoint for *F. nucleatum* is ≤0.5 ng/µL for EUCAST and ≤4 ng/µL for CLSI, while for *P. intermedia* it is ≤0.25 ng/µL for EUCAST and ≤4 ng/µL for CLSI. ^d^ The MTZ susceptibility breakpoint for *F. nucleatum* is ≤0.5 ng/µL for EUCAST and ≤8 ng/µL for CLSI, while for *P. intermedia* it is ≤4 ng/µL for EUCAST and ≤8 ng/µL for CLSI. ^e^ The AMX-EUCAST susceptibility zone diameter breakpoint for *F. nucleatum* is ≥23 mm, while for *P. intermedia* it is ≥24 mm. ^f^ The MTZ-EUCAST susceptibility zone diameter breakpoint for *F. nucleatum* is ≥30 mm, while for *P. intermedia* it is ≥22 mm.

## Data Availability

The original contributions presented in this study are included in the article. Further inquiries can be directed to the corresponding authors.
